# Agonist Binding Reshapes the Kinetic Landscape and Allosteric Communication of APLNR Toward Activation-Competent States

**DOI:** 10.3390/ijms27177674

**Published:** 2026-08-27

**Authors:** Hong Dai, Jun-Yao Zhu, Bao-Dan Zhang, Meng-Ting Liu, Peng Sang, Li-Quan Yang

**Affiliations:** 1 College of Agriculture and Biological Science, Dali University, Dali 671003, China; daidai@dali.edu.cn (H.D.); zhujunyao@stu.dali.edu.cn (J.-Y.Z.); 18687047890@163.com (B.-D.Z.); lminimt@163.com (M.-T.L.); 2 Co-Innovation Center for Cangshan Mountain and Erhai Lake Integrated Protection and Green Development of Yunnan Province, Dali University, Dali 671003, China; 3 Key Laboratory of Bioinformatics and Computational Biology, Department of Education of Yunnan Province, Dali University, Dali 671003, China; 4 College of Pharmacy, Dali University, Dali 671003, China

**Keywords:** G protein-coupled receptors (GPCRs), apelin receptor (APLNR), conformational dynamics, allosteric communication, signaling competence, Markov state models (MSMs), neural relational inference (NRI)

## Abstract

APLNR is a therapeutically important G protein-coupled receptor (GPCR) implicated in cardiovascular and metabolic regulation; however, how agonist binding reorganizes receptor dynamics to promote signaling competence remains poorly understood. Here, we used the small-molecule agonist CMF-019 as a representative ligand and integrated Gaussian accelerated molecular dynamics (GaMD), Markov state models (MSMs), and neural relational inference (NRI) to characterize the conformational dynamics, kinetic organization, and allosteric communication of APLNR in apo and CMF-019-bound states. We found that CMF-019 binding altered structural flexibility in extracellular and intracellular regions while modifying collective motions within the transmembrane core, indicating a transition toward a more signaling-permissive dynamic state. MSM analyses further revealed that CMF-019 binding redistributed APLNR toward activation-related intermediate conformations and accelerated transitions among metastable states. Mechanistically, NRI uncovered extensive rewiring of the receptor communication network, in which CMF-019 binding strengthened transmembrane coupling and redirected signal propagation toward more convergent signaling routes linked to intracellular functional regions. Together, these findings suggest that CMF-019 promotes APLNR signaling competence through integrated kinetic and allosteric remodeling, revealing a dynamic mechanism by which an agonist can reorganize receptor communication prior to downstream coupling.

## 1. Introduction

The apelin receptor (APLNR, also known as APJ) is a class A G protein-coupled receptor (GPCR) that plays pivotal roles in cardiovascular homeostasis, angiogenesis, metabolic regulation, and fluid balance [1,2,3,4]. Dysregulation of APLNR signaling has been implicated in diverse pathological conditions, including heart failure, pulmonary hypertension, metabolic disorders, and tumor progression, positioning APLNR as an increasingly attractive therapeutic target [2,3,4,5]. The receptor can be activated by endogenous agonists such as Apelin and Elabela, as well as by an expanding range of small-molecule agonists. These chemically and structurally diverse ligands differ in molecular size, chemical composition, binding mode, and receptor contacts, raising the possibility that they may stabilize distinct receptor conformational ensembles and signaling profiles. Pharmacological studies of biased APLNR ligands further support the concept that ligand binding can differentially modulate receptor signaling rather than simply producing a single uniform active state [5,6,7,8]. Nevertheless, an important mechanistic question remains unresolved: how agonist binding alone prepares APLNR for signaling prior to downstream coupling.

**Figure 1:**
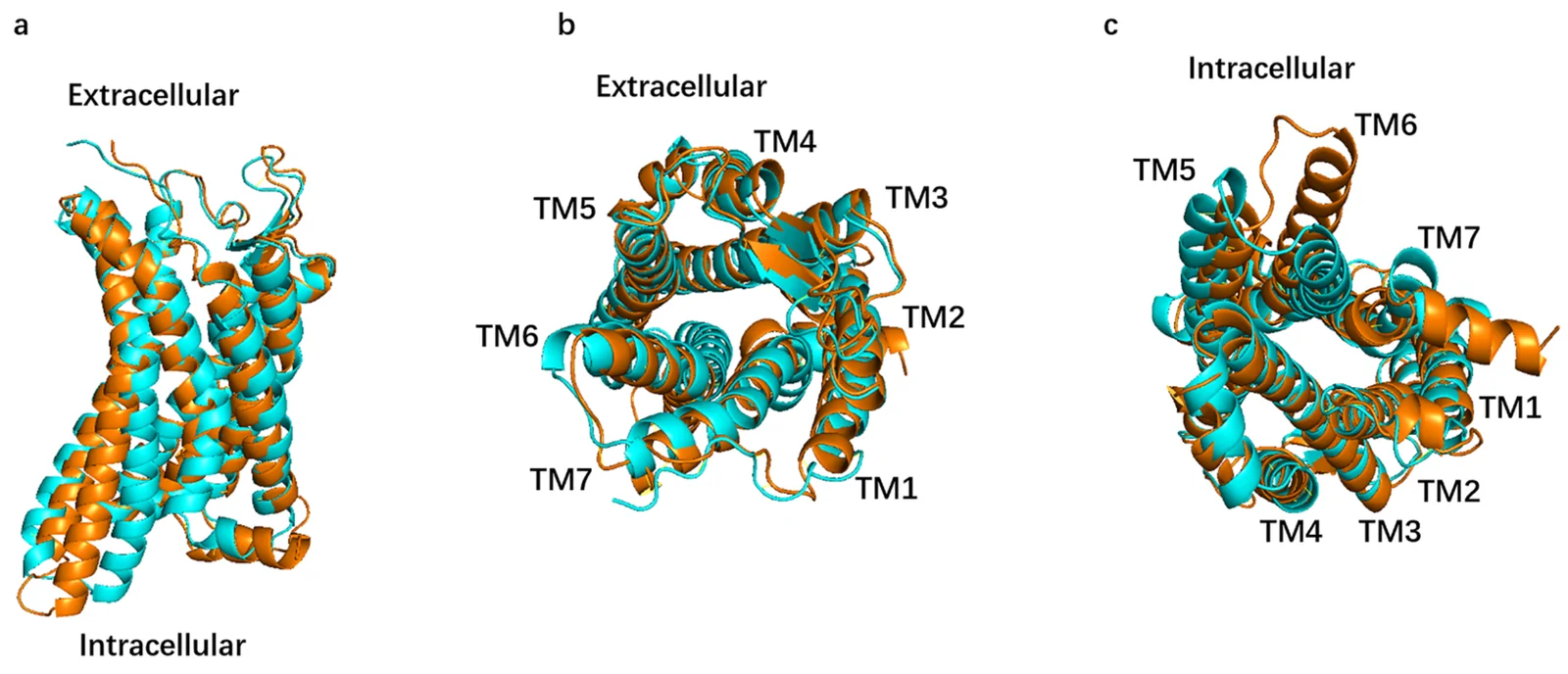
Structural comparison of inactive and active conformations of APLNR. (**a**) Superposition of the experimentally resolved inactive (cyan; PDB ID: 8XQJ) and active (orange; PDB ID: 8XZH) structures of APLNR. (**b**) Extracellular view of the superimposed receptor structures, illustrating ligand-facing conformational rearrangements among transmembrane helices (TM1–TM7). (**c**) Intracellular view of the receptor highlighting substantial outward displacement and reorientation of TM6 in the active conformation.

Canonical GPCR activation is generally associated with large-scale conformational rearrangements, most notably the outward displacement of transmembrane helix 6 (TM6), which creates an intracellular cavity competent for G protein engagement [9,10,11,12]. Structural comparison between experimentally resolved inactive and active APLNR conformations similarly reveals substantial rearrangements across the receptor, particularly within intracellular signaling regions (Figure 1a–c). In the active-state structure, the intracellular end of TM6 undergoes pronounced outward movement relative to the inactive conformation, consistent with activation mechanisms broadly conserved among class A GPCRs. However, accumulating evidence suggests that agonist binding alone is frequently insufficient to fully stabilize a canonical active-state architecture, and that complete receptor activation often requires additional stabilization by intracellular effectors such as G proteins or arrestins [10,11,12,13,14]. These observations raise an important possibility: rather than directly locking APLNR into a single active conformation, agonist binding may instead reorganize the receptor into a signaling-permissive conformational ensemble that facilitates downstream coupling.

Increasing evidence suggests that GPCR signaling is fundamentally dynamic rather than static, emerging through conformational ensembles composed of transient intermediates, stochastic transitions, and distributed communication across distant structural regions [10,12,15,16,17]. Although cryo-electron microscopy and X-ray crystallography have provided invaluable snapshots of APLNR in ligand-bound and inactive conformations, static structures alone remain insufficient to explain how agonist binding reshapes receptor behavior prior to downstream engagement [6,7,8]. This limitation is particularly important for APLNR, where the molecular basis by which agonist binding reorganizes receptor dynamics remains poorly understood. In particular, whether ligand engagement directly stabilizes an active-state conformation or instead redistributes the receptor across activation-compatible intermediate conformations remains unresolved.

Importantly, receptor activation is not governed solely by local structural rearrangements but also by the emergence of integrated communication across distant regions of the protein [18,19,20]. Increasing evidence indicates that GPCR signaling depends on dynamically coupled residue networks that transmit information from extracellular ligand-binding sites to intracellular effector-binding interfaces through transient allosteric pathways [18,19,20,21]. Such long-range communication is inherently dynamic, frequently involving distributed and metastable interactions that cannot be readily resolved through static structural comparison alone. For APLNR, however, whether agonist binding reorganizes these hidden communication networks—and how such reorganization contributes to signaling competence—remains largely unexplored.

Addressing this question requires more than structural comparison alone. The emergence of receptor signaling competence is inherently a multiscale dynamical process involving conformational redistribution, kinetic transitions, and long-range communication. Although conventional molecular dynamics simulations have provided important insights into GPCR activation, they often remain insufficient to simultaneously resolve conformational ensembles, transition kinetics, and transient communication pathways that collectively govern receptor function [15,16,17]. Recent advances in enhanced sampling, kinetic modeling, and graph-based inference provide an opportunity to bridge this gap. Gaussian accelerated molecular dynamics (GaMD) enables efficient exploration of conformational landscapes, Markov state models (MSMs) resolve metastable states and transition kinetics, and neural relational inference (NRI) enables inference of hidden dynamic couplings underlying long-range communication [22,23,24,25].

Here, we sought to determine how binding of the small-molecule agonist CMF-019 reshapes the conformational and communication landscape of APLNR prior to downstream coupling. CMF-019 was selected as a representative agonist because its activity at APLNR has been experimentally characterized and structural information is available for ligand-induced receptor activation. By integrating GaMD, MSMs, and NRI, we systematically investigated the dynamic ensembles, kinetic organization, and long-range communication patterns of APLNR in the apo and CMF-019-bound states. Rather than assuming receptor activation as a simple structural transition, we specifically asked whether CMF-019 binding promotes APLNR signaling competence through integrated kinetic and allosteric remodeling [10,11,14,20]. This approach provides a dynamic framework for understanding how a representative agonist prepares APLNR for downstream signaling while providing a basis for considering ligand-dependent mechanisms of receptor activation.

## 2. Results

### 2.1. CMF-019 Binding Selectively Constrains Receptor Flexibility While Preserving Collective Motions

**Figure 2:**
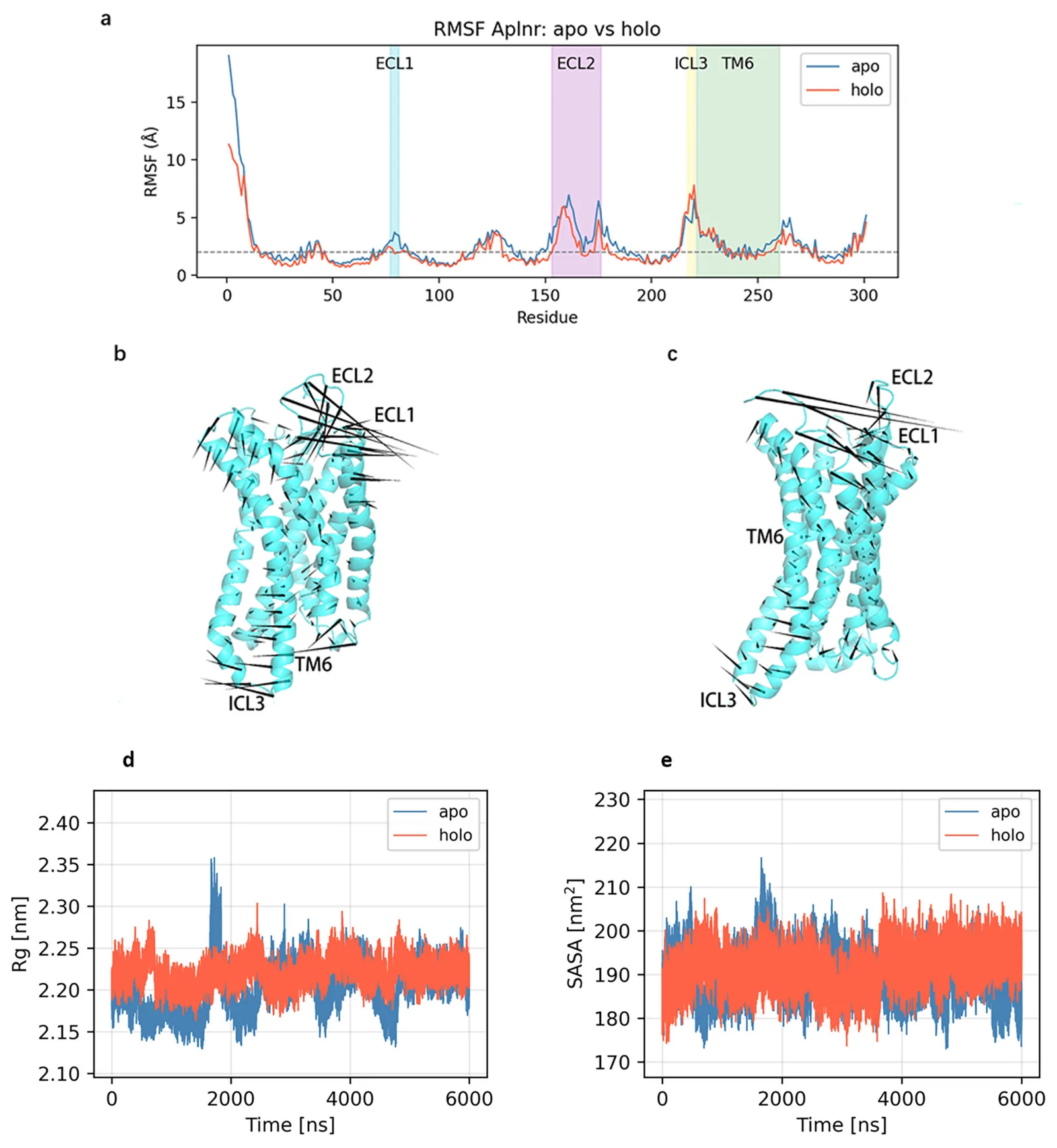
CMF-019-induced reorganization of APLNR structural flexibility and collective motions. (**a**) Comparison of residue-level root-mean-square fluctuations (RMSFs) between the apo (blue) and holo (red) states of APLNR. The dashed line indicates the average RMSF level across the receptor. Shaded regions highlight representative structural elements exhibiting altered flexibility upon ligand binding. (**b**) Porcupine plot illustrating dominant collective motions of APLNR in the apo state. (**c**) Porcupine plot of the holo state. Black arrows indicate the direction and relative amplitude of dominant residue displacements along the principal collective motion mode. (**d**) Radius of gyration (Rg) and (**e**) solvent-accessible surface area (SASA) of apo and holo APLNR.

To determine whether CMF-019 binding simply stabilizes the APLNR structure or instead reorganizes its dynamic behavior, we first characterized receptor flexibility and collective motions in the apo and CMF-019-bound states using RMSD, RMSF, and principal component analysis (PCA). Although both states rapidly reached conformational equilibration during the early stage of simulation (Figure 2 and Figure S1), their dynamic behaviors diverged substantially. Compared with the CMF-019-bound state, the apo receptor displayed broader RMSD fluctuations, indicating greater conformational variability. In contrast, the CMF-019 binding constrained overall structural fluctuations and narrowed the conformational space sampled by APLNR, suggesting that ligand engagement modulates receptor dynamics while preserving the overall structural architecture of APLNR [10,11,20].

Residue-level flexibility analysis further revealed that ligand-induced dynamic remodeling occurred in a region-specific manner rather than through uniform rigidification (Figure 2a) [11,20]. In the apo state, extracellular regions—particularly ECL1 and ECL2—exhibited elevated fluctuations, indicative of substantial conformational heterogeneity in the absence of ligand. Upon CMF-019 binding, fluctuations in these extracellular loops were markedly suppressed, whereas enhanced mobility remained evident around ICL3 and the intracellular end of TM6, regions closely associated with GPCR activation and downstream effector coupling [11,14]. Notably, the extracellular N-terminus remained highly dynamic in both states, consistent with the intrinsically flexible characteristics commonly observed in GPCR extracellular regions. Together, these observations suggest that CMF-019 binding selectively reduces structural flexibility in extracellular regions while preserving localized intracellular flexibility associated with signaling-related conformational rearrangements.

To further resolve the dominant motions underlying receptor dynamics, PCA and porcupine analyses were performed to visualize collective conformational displacements (Figure 2b,c). In the apo state, APLNR sampled extensive conformational motions across extracellular and intracellular regions, accompanied by pronounced displacements near ICL3 and TM6, reflecting a highly flexible conformational ensemble. By contrast, CMF-019 binding substantially reduced the amplitude of large-scale stochastic motions and imposed greater conformational constraint on the transmembrane core. Importantly, however, collective motions within activation-related regions were not abolished. Characteristic displacements remained evident near TM6 and intracellular regions implicated in receptor activation, suggesting that ligand binding selectively filters conformational fluctuations while preserving collective motions potentially relevant to signaling competence [10,11,14].

The global structural properties of APLNR were further assessed using the radius of gyration (Rg) and solvent-accessible surface area (SASA) (Figure 2d,e). The Rg profiles of apo and holo APLNR largely overlapped throughout the 6 μs simulations, although the holo receptor exhibited a modestly higher average Rg. Similarly, the holo state showed a slightly higher SASA than the apo state, while substantial overlap between the two trajectories was observed. The modest increase in Rg and SASA suggests that CMF-019 binding does not promote global receptor compaction, but is associated with a slightly more expanded and spatially redistributed receptor ensemble.

Together, these findings indicate that the CMF-019-bound state does not simply rigidify APLNR into a static conformation. Instead, ligand engagement selectively reorganizes receptor dynamics, shifting APLNR toward a more constrained yet functionally organized conformational ensemble [10,11,20]. However, if CMF-019 binding does not merely stabilize the receptor, an immediate mechanistic question emerges: what conformational states are actually being preferentially populated?

### 2.2. CMF-019 Induces Localized Structural Remodeling of APLNR

**Figure 3:**
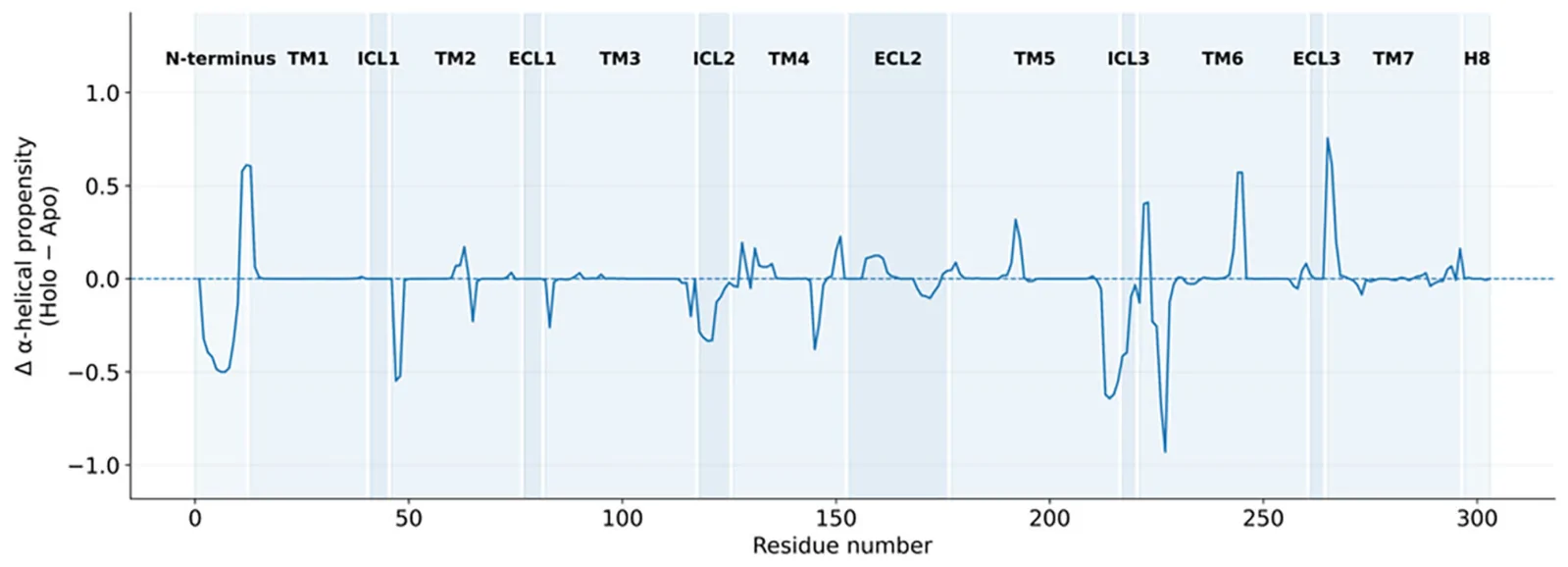
CMF-019-induced changes in α-helical propensity of APLNR. Residue-level changes are shown as Δ α-helical propensity = holo − apo. Shaded regions indicate the structural topology of APLNR.

To further characterize ligand-induced structural rearrangements, we analyzed residue-level changes in α-helical propensity and the TM3–TM6 distance between apo and holo APLNR (Figure 3 and Figure S2). The Δ α-helical propensity (holo − apo) analysis showed that the overall α-helical architecture of APLNR was largely preserved, whereas localized changes were observed in several regions. Notably, pronounced alterations were detected around ICL3 and TM6, with both decreases and increases in α-helical propensity, indicating heterogeneous local remodeling rather than global disruption of the receptor helical scaffold.

We next monitored the TM3–TM6 distance (R3.50–L6.34) as a structural descriptor of the intracellular receptor core [14]. Both apo and holo APLNR exhibited substantial temporal fluctuations, indicating that the receptor dynamically sampled multiple TM3–TM6 configurations throughout the simulations [10,11,20]. The CMF-019 binding displayed repeated excursions toward larger TM3–TM6 distances during the simulation, together with transient contractions, suggesting that CMF-019 binding modulates the dynamic accessibility of alternative intracellular conformations rather than locking the receptor into a single open state [10,11,20].

Together, these analyses indicate that CMF-019 binding preserves the global helical framework of APLNR while promoting localized structural remodeling and altering the conformational dynamics of the intracellular receptor core. These structural changes provide a basis for the distinct conformational states and kinetic properties identified by the subsequent MSM analysis.

### 2.3. CMF-019 Binding Reshapes the Conformational and Kinetic Landscape of APLNR

The ligand-induced reorganization of receptor dynamics observed above raises a fundamental mechanistic question: does CMF-019 binding preferentially stabilize the APLNR structure toward a canonical active conformation, or instead reshape the conformational ensemble without fully completing receptor activation? To address this question, we constructed Markov state models (MSMs) from GaMD trajectories to characterize the conformational ensemble and kinetic organization of receptor states [17,23,26]. Model robustness was supported by microstate optimization, lag-time selection, and Chapman–Kolmogorov validation, indicating that the resulting MSMs reliably captured the dominant conformational transitions sampled by APLNR (Figures S3–S5).

**Figure 4:**
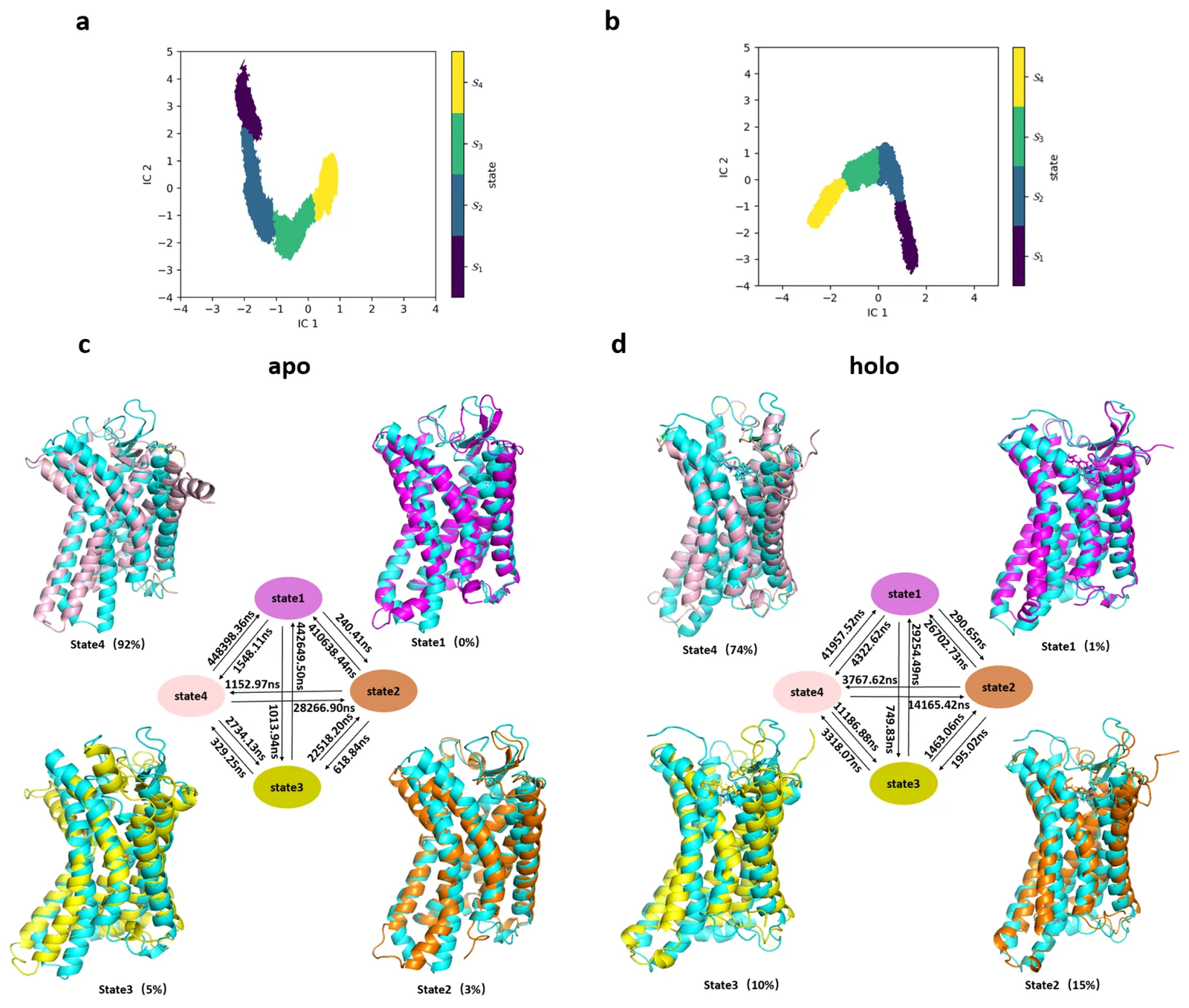
CMF-019 binding reshapes the kinetic landscape of APLNR toward activation-compatible intermediate conformations. (**a**,**b**) Projection of APLNR conformational ensembles onto the first two time-lagged independent components (IC1 and IC2) derived from Markov state models (MSMs) for the apo (**a**) and CMF-019-bound (**b**) states. Four metastable conformational states (S1–S4) were identified and color-coded according to state assignment. (**c**,**d**) MSM-derived transition networks and representative structures of the metastable conformational states for the apo (**c**) and CMF-019-bound (**d**) states. For each corresponding metastable state, the MSM-derived representative structures are superimposed with the corresponding initial receptor structure to facilitate comparison of global and local conformational features. The cyan structure represents the initial receptor conformation, whereas the non-cyan structure represents the MSM-derived representative conformation. Arrows indicate dominant transition pathways between macrostates, and numbers denote mean first passage times (MFPTs). The percentages below the representative structures indicate the corresponding state populations.

To quantify activation-related conformational rearrangements, we monitored the intracellular displacement of TM6 using the distance between Arg111^3.50^ (TM3) and Leu231^6.34^ (TM6), a structural hallmark widely associated with GPCR activation (Figure 4) [9,11,14]. The experimentally resolved active-state APLNR structure (PDB ID: 8XZH) was used as a structural reference. Despite the ligand-induced changes in structural flexibility and collective motions observed in the CMF-019-bound state, CMF-019 binding alone did not result in sustained adoption of a canonical active-state architecture. Instead, both apo and CMF-019-bound APLNR predominantly sampled conformations that remained within inactive-like to activation-compatible structural regimes, rather than converging on the canonical active state, indicating that receptor activation remained incomplete in the absence of downstream coupling partners [9,10,14].

This observation was particularly unexpected because the ligand-bound receptor exhibited distinct changes in structural flexibility and collective motions relative to the apo state. Under a conventional activation model, such ligand-induced dynamic changes would be anticipated to favor convergence toward an active-state conformation. However, the MSM results suggest otherwise: CMF-019 binding alone appears insufficient to lock APLNR into a fully active receptor state. Rather than converging toward a single activated architecture, ligand binding reshaped the receptor into a structurally constrained yet conformationally heterogeneous ensemble [12,20]. More specifically, APLNR in the apo state overwhelmingly occupied a dominant conformational basin (State4, 92%), whereas alternative states remained sparsely populated (Figure 4c). By contrast, CMF-019 binding substantially redistributed the conformational ensemble. In APLNR in the CMF-019-bound state, the dominance of the major basin decreased (State4, 74%), accompanied by marked increases in the population of metastable intermediate states (State2, 15%; State3, 10%) (Figure 4d). Notably, none of these dominant conformations fully converged toward the experimentally resolved active-state structure. Instead, CMF-019 binding shifted APLNR toward a broader ensemble of metastable, activation-compatible intermediate conformations.

If CMF-019 binding alone does not fully stabilize receptor activation, an immediate mechanistic inconsistency emerges: why does signaling nevertheless become more favorable? To address this, we examined transition kinetics among metastable states using mean first passage time (MFPT) analysis derived from the MSM framework. In the apo state, APLNR remained predominantly trapped within a highly stable conformational basin (State4), from which transitions to alternative states occurred only infrequently. The prolonged return times from intermediate conformations to the dominant basin suggest that ligand-free APLNR is kinetically confined within a restricted region of conformational space, limiting efficient exploration of activation-related states (Figure 4c) [12,17,20].

By contrast, CMF-019 binding substantially reorganized the kinetic landscape of APLNR. In the CMF-019-bound state, transitions among metastable states became markedly more frequent, with substantially reduced MFPTs between activation-related intermediates (Figure 4d). Although a dominant basin remained present, the kinetic confinement observed in the apo state was partially relieved, enabling more efficient transitions across alternative conformational states. Notably, transition rates from the dominant basin toward intermediate conformations accelerated by approximately one order of magnitude relative to the apo state, indicating that CMF-019 binding enhances receptor signaling potential not through structural fixation, but through kinetic redistribution across activation-related states [10,11,12,14,20].

Together, these findings support an alternative mechanism for agonist-driven signaling in APLNR [10,11,12,14]. Rather than directly stabilizing a fully active receptor conformation, CMF-019 binding appears to release the receptor from kinetic trapping, reshaping signaling competence through enhanced conformational accessibility among metastable intermediates [10,11,12,14,20]. Yet one mechanistic problem remains unresolved: how are these redistributed conformational transitions coupled across distant regions of the receptor to enable efficient signal propagation?

### 2.4. CMF-019 Binding Rewires Long-Range Communication Within APLNR

**Figure 5:**
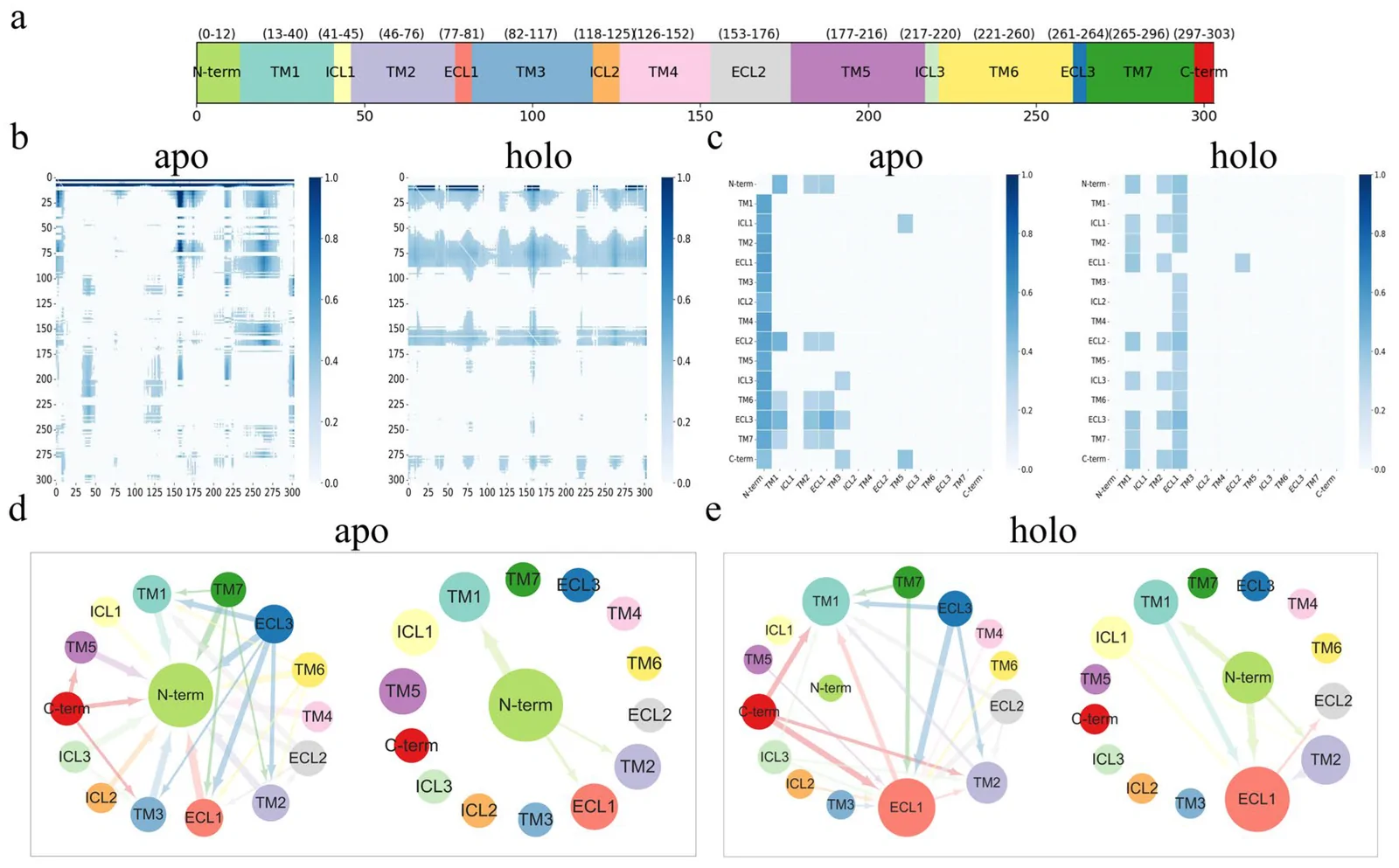
CMF-019 binding rewires the long-range communication network of APLNR. (**a**) Domain segmentation scheme of APLNR used for neural relational inference (NRI) analysis. The receptor was divided into major structural modules, including the N-terminus, transmembrane helices (TM1–TM7), intracellular loops (ICLs), extracellular loops (ECLs), and the C-terminus. (**b**) Residue-level interaction matrices inferred by NRI for the apo and CMF-019-bound states. Color intensity represents inferred interaction strength between residue pairs. (**c**) Domain-level interaction matrices summarizing long-range communication between structural modules. (**d**,**e**) Domain communication networks inferred from NRI for the apo (**d**) and CMF-019-bound (**e**) states. Node size reflects relative communication importance, and edge thickness represents interaction strength.

The kinetic redistribution observed above suggests that binding enhances conformational accessibility among activation-related states. However, altered transition kinetics alone cannot explain how these distributed conformational changes become coupled across the receptor. If signaling competence emerges in the CMF-019-bound state, what organizes these transitions into a functionally coherent response? To address this question, we employed neural relational inference (NRI) to characterize CMF-019-induced remodeling of residue–residue and domain–domain communication networks within APLNR (Figure 5a) [24,25].

NRI-derived interaction matrices revealed a substantial reorganization of long-range coupling patterns following CMF-019 binding (Figure 5b,c) [18,19,25]. In the apo state, interaction strengths were generally weak and distributed diffusely across domains, with relatively limited direct coupling among transmembrane helices and extracellular regions. By contrast, CMF-019 binding markedly strengthened interactions throughout the receptor, particularly among extracellular loops and transmembrane segments. Notably, coupling between ECL1 and multiple transmembrane helices—including TM2, TM3, and TM7—became substantially enhanced in the holo state, suggesting that extracellular ligand engagement induces a reorganization of dynamic coupling patterns across the receptor rather than a purely local structural response [9,10,11,18,19].

To further resolve how these interactions are organized at the network level, we reconstructed directed domain–domain communication networks from the NRI-derived coupling matrices (Figure 5d,e) [24,25]. In the apo state, the network adopted a relatively diffuse architecture dominated by the N-terminus, through which most inter-domain interactions were indirectly routed. Direct communication among transmembrane regions remained comparatively sparse, reflecting a loosely organized signaling architecture consistent with the kinetically restricted conformational landscape described above [18,19]. In contrast, CMF-019 binding induced a pronounced network reorganization. The dominant role of the N-terminus diminished substantially, while ECL1 emerged as a new communication hub, mediating strengthened coupling among extracellular and transmembrane regions. Simultaneously, direct interactions between transmembrane helices became markedly reinforced, transforming the receptor from a loosely organized network into a more integrated and densely connected communication architecture.

Importantly, this reorganization provides a mechanistic explanation for the altered kinetic behavior observed in the CMF-019-bound state [12,17,20,25]. Rather than functioning as isolated structural fluctuations, agonist-induced conformational transitions appear to become increasingly coupled through strengthened long-range coupling across the receptor [9,10,11,18,19]. In this view, signaling competence may emerge not only through enhanced kinetic accessibility, but also through progressive allosteric rewiring that organizes distributed receptor motions into a more coherent communication framework. Yet a critical mechanistic uncertainty remains unresolved: does this rewired network genuinely enhance signal propagation, or merely redistribute dynamic coupling without improving communication efficiency?

### 2.5. CMF-019 Binding Promotes Convergent Allosteric Communication Toward Signaling Regions

**Figure 6:**
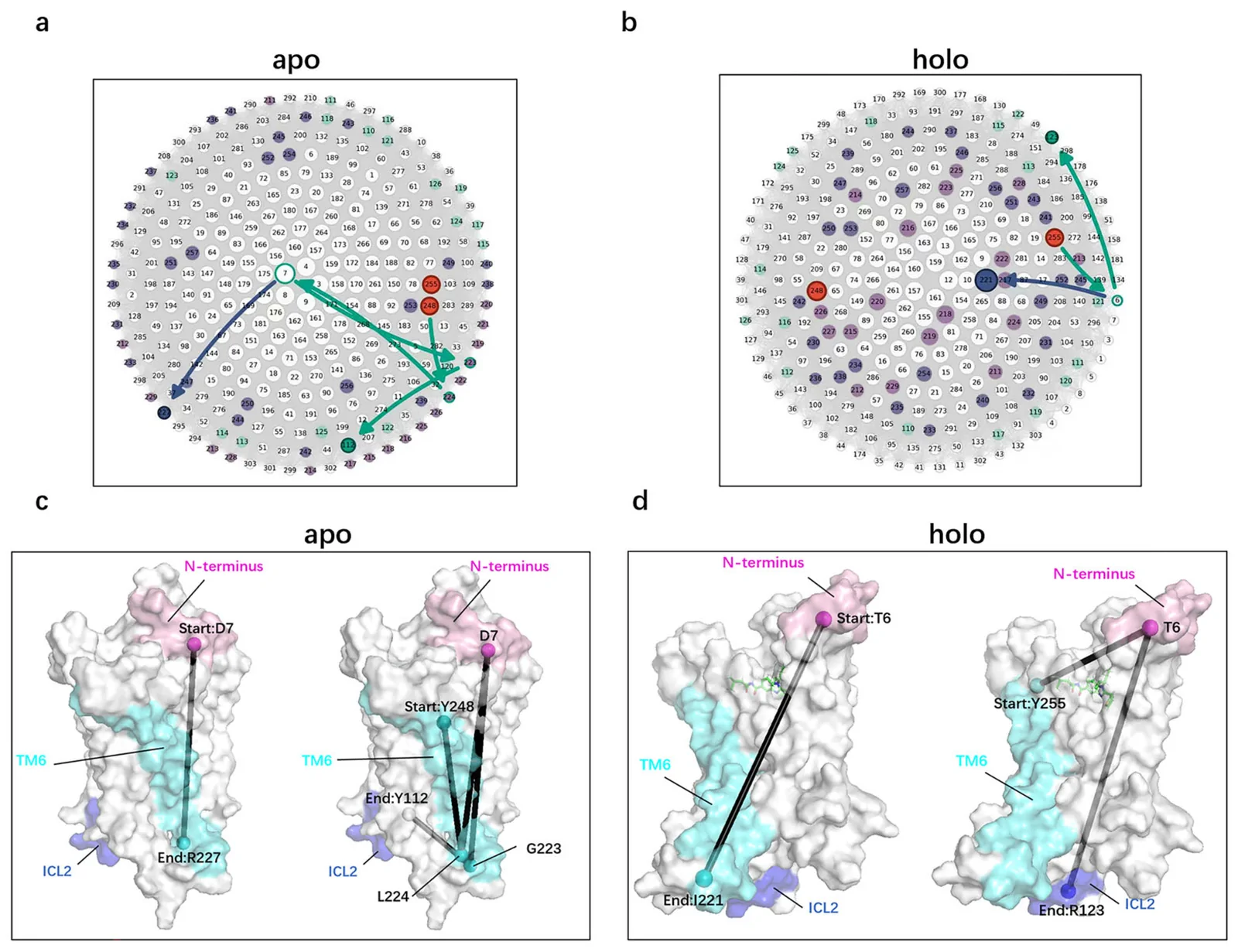
CMF-019 binding redirects allosteric signal propagation toward convergent intracellular communication pathways in APLNR. (**a**,**b**) Concentric network representations of the shortest allosteric communication pathways in the apo (**a**) and CMF-019-bound (**b**) states. Nodes represent residues and are arranged according to network connectivity, while blue-gray and green edges represent the dominant shortest communication routes. (**c**,**d**) Structural mapping of the shortest allosteric communication pathways onto the receptor surface for the apo (**c**) and CMF-019-bound (**d**) states. The N-terminus is shown in pink, TM6 in cyan, and ICL2 in blue.

The extensive allosteric rewiring observed above suggests that agonist binding reorganizes dynamic coupling patterns across APLNR. Yet a critical mechanistic question remains unresolved: does this rewired network genuinely enhance signal propagation, or merely redistribute internal interactions without improving communication efficiency? To resolve this question, we employed shortest-path analysis using Dijkstra’s algorithm to characterize long-range information flow from the extracellular ligand-sensing region toward intracellular signaling interfaces, with particular emphasis on communication routes connecting the N-terminus to TM6 and ICL2, two regions closely associated with GPCR activation and downstream effector engagement (Figure 6a–d) [14,19,20,27].

In the apo state, communication pathways remained broadly distributed across the receptor network, lacking a clearly dominant route for signal transmission (Figure 6a,c). Signal propagation toward intracellular regions followed multiple dispersed trajectories, indicative of weakly integrated and inefficient long-range communication. Consistent with the diffuse network organization identified by NRI, communication in the ligand-free receptor appeared fragmented, with information flow distributed across structurally heterogeneous routes rather than concentrated into a coherent signaling architecture.

By contrast, CMF-019 binding profoundly reorganized signal propagation within APLNR (Figure 6b,d). In the CMF-019-bound state, shortest paths became markedly more centralized, converging into dominant communication routes extending from extracellular sensing regions toward intracellular signaling interfaces. Rather than propagating diffusely, signal transmission became increasingly funneled through convergent pathways enriched around TM6 and ICL2, suggesting enhanced coupling between extracellular ligand recognition and intracellular functional regions. Importantly, several residues located within these convergence zones emerged as putative relay nodes, suggesting that CMF-019 binding promotes not only stronger interactions, but also more organized and directed information flow across the receptor.

This transition from diffuse communication to convergent signal propagation provides a mechanistic explanation for how agonist binding enhances signaling competence despite the absence of a fully stabilized active-state conformation. Rather than directly locking APLNR into a canonical active architecture, CMF-019 binding appears to kinetically and allosterically reorganize the receptor into a communication state in which information flow toward intracellular signaling regions becomes increasingly favored. In this framework, receptor activation emerges not through structural fixation, but through progressive reorganization of dynamic accessibility, long-range coupling, and allosteric signal propagation [10,14,19,20,27].

## 3. Discussion

GPCR activation is increasingly understood as a dynamic process emerging from organized conformational ensembles rather than a simple transition between inactive and active structures [9,10,11,14]. Within this framework, our findings suggest that agonist-driven APLNR activation is best understood not as immediate structural conversion, but as progressive dynamic reorganization. CMF-019 binding altered structural flexibility and collective receptor motions and shifted the conformational ensemble toward activation-related intermediate states distributed across the receptor landscape.

This observation suggests that agonist binding acts less as a structural “switch” and more as a dynamic organizer that reshapes receptor behavior toward signaling permissiveness. Rather than enforcing occupation of a single structural endpoint, agonist binding appears to selectively constrain specific conformational fluctuations while preserving motions associated with activation-related regions. Such behavior aligns with emerging views that GPCR efficacy often arises from redistribution of conformational populations rather than stabilization of one dominant active architecture [9,10,11,13,14]. Yet dynamic reorganization alone does not explain why signaling becomes increasingly favorable, raising an immediate mechanistic question: how does agonist binding reshape the receptor landscape to facilitate productive signaling?

An increasing body of evidence suggests that GPCR signaling is governed not only by structural states, but also by the kinetics controlling transitions between metastable conformations [10,14,16,26]. Consistent with this view, our MSM analyses indicate that CMF-019 reshapes APLNR signaling competence through kinetic redistribution rather than structural fixation. In the apo state, APLNR remained predominantly confined within a dominant conformational basin, suggesting restricted access to signaling-related conformations. By contrast, agonist binding redistributed the conformational ensemble and accelerated transitions among metastable intermediates, partially relieving kinetic confinement and enhancing accessibility to activation-related states. Rather than locking APLNR into a single activated endpoint, CMF-019 appears to release the receptor from kinetic trapping, thereby enabling more efficient exploration of signaling-relevant conformations.

Yet enhanced conformational accessibility alone remains insufficient to explain productive receptor signaling. Signaling requires not only access to favorable states, but also coordination among distributed receptor motions across distant structural regions [19,20,27].

Accumulating evidence suggests that GPCR activation emerges through integrated long-range communication networks that dynamically organize information flow across the receptor [19,20,27]. Within this framework, our NRI analyses suggest that agonist binding reorganizes APLNR not simply by strengthening residue interactions, but by fundamentally rewiring its dynamic coupling architecture. In the apo state, receptor communication remained relatively diffuse, with interactions primarily routed through a loosely connected N-terminus-centered network. In contrast, agonist binding induced pronounced long-range coupling among transmembrane helices and established ECL1 as a communication hub, linking extracellular sensing regions with the receptor core. Rather than reflecting isolated local rearrangements, these findings suggest that agonist binding progressively transforms distributed receptor motions into a more coherent signaling framework.

However, network rewiring alone does not necessarily ensure productive signaling. If agonist binding reorganizes receptor communication, an important mechanistic question remains: does this rewired network truly improve signal propagation toward intracellular signaling regions?

Effective receptor activation ultimately depends on whether signaling information can be efficiently transmitted toward intracellular functional regions. Consistent with emerging views that GPCR efficacy depends on long-range communication dynamics [19,20,21,25,27], our shortest-path analyses suggest that agonist binding transforms the rewired APLNR network into a more efficient signaling architecture. In the apo state, signaling routes remained broadly distributed across diffuse pathways, indicative of weakly integrated information transfer. By contrast, agonist binding reorganized signal propagation into dominant routes converging toward TM6 and ICL2, two regions closely associated with intracellular signaling and effector engagement [14,20]. Rather than propagating through fragmented trajectories, signaling information in the holo receptor became increasingly funneled toward signaling-relevant regions, indicating that agonist binding progressively organizes distributed receptor motions into directed information flow toward signaling-relevant regions.

This transition from diffuse communication to convergent signal propagation provides a mechanistic explanation for how APLNR becomes increasingly signaling-permissive despite the absence of a fully stabilized activated endpoint.

Taken together, our findings support a kinetic–allosteric priming model for agonist-driven APLNR activation. Rather than directly stabilizing a single activated structure, CMF-019 progressively reorganizes receptor behavior through a multistep process involving dynamic reorganization, kinetic redistribution, allosteric rewiring, and convergent signal propagation. Within this framework, APLNR activation emerges not through immediate structural completion, but through progressive establishment of a signaling-permissive ensemble that facilitates downstream signaling engagement [10,11,14]. Although downstream effectors were not explicitly modeled in the present study, our findings suggest that agonist binding alone may already establish the dynamic prerequisites required for productive signaling. More broadly, this work provides a mechanistic framework for understanding how APLNR ligand efficacy may emerge through kinetic and allosteric reorganization rather than structural fixation, while recognizing that these mechanisms may vary across different ligands [8,28].

An important consideration is that the conformational reshaping observed in this study may be ligand-dependent. APLNR is activated by endogenous peptide agonists, including Apelin and Elabela, as well as structurally diverse ligands [3,4,6,28,29]. These ligands differ substantially in molecular size, chemical composition, binding mode, and receptor contacts, and therefore may stabilize distinct receptor conformational ensembles [6,28,29]. In particular, pharmacological studies have demonstrated that structurally distinct APLNR ligands can exhibit biased signaling properties, supporting the concept that ligand binding may differentially reshape receptor dynamics and downstream signaling [5,8,28]. For example, (-)-epicatechin has been reported as a biased ligand of APLNR, providing experimental evidence that chemically distinct ligands can differentially modulate receptor signaling [8]. In this context, our results should be interpreted specifically as describing the dynamic effects of CMF-019 binding rather than as establishing a universal activation mechanism shared by all APLNR agonists. Future studies will be needed to identify common versus ligand-specific conformational and allosteric features through comparative simulations and experimental characterization of endogenous peptides and structurally diverse agonists.

## 4. Materials and Methods

### 4.1. System Preparation and Enhanced Conformational Sampling

The inactive-state structure of the apelin receptor (APLNR) was obtained from the experimentally resolved crystal structure deposited in the Protein Data Bank (PDB ID: 8XQJ) [29], which served as the initial template for all simulations. Prior to system construction, crystallographic water molecules and non-protein heteroatoms were removed, missing hydrogen atoms were added, and residue completeness was verified. Protonation states of ionizable residues were assigned under physiological pH conditions. For the apo state, only the receptor structure was retained. To generate the CMF-019-bound state, the small-molecule agonist CMF-019 [28], previously reported as an APLNR agonist, was selected as a representative ligand and modeled and docked into the orthosteric binding pocket using AutoDock Vina 1.5.7 [30]. Hereafter, “holo” specifically refers to the CMF-019-bound state and is used interchangeably with “CMF-019-bound” where appropriate. Docking was performed based on the experimentally resolved receptor structure, with the orthosteric site defined as the docking region. The receptor–ligand complex exhibiting the lowest binding energy and a structurally reasonable binding pose was selected as the starting conformation for subsequent simulations.

To preserve the native membrane environment and capture receptor dynamics under physiologically relevant conditions, both apo and holo states were embedded in a mixed lipid bilayer composed of DOPE:DOPG (3:1 molar ratio) [31] using the PACKMOL-Memgen workflow implemented in AMBER Tools 25 [32]. The membrane systems were explicitly solvated, and K^+^/Cl^−^ ions were added using the tleap module to neutralize the molecular systems and achieve a final ionic concentration of 0.15 M. Protein dynamics were described using the ff14SB [33] force field, membrane lipids using Lipid21 [34], and water molecules using the TIP3P model [35]. Ligand parameters were generated using the GAFF2 force field with atomic charges assigned using the AM1-BCC method, and missing parameters were supplemented using parmchk2 [36,37]. To improve simulation stability, hydrogen mass repartitioning (HMR) was applied to enable a stable 2 fs integration timestep [38]. AMBER topology and coordinate files were subsequently generated for all molecular simulation systems.

To obtain equilibrated receptor ensembles suitable for long-timescale conformational sampling, all systems underwent a multistage relaxation procedure using the GPU-accelerated pmemd.cuda engine in AMBER25 [39,40,41]. System minimization was conducted in three sequential stages: (1) minimization of solvent molecules and ions with positional restraints of 50.0 kcal mol^−1^ Å^−2^ applied to solute heavy atoms; (2) minimization with protein side chains released while maintaining backbone restraints; and (3) unrestrained minimization of the entire system. The minimized systems were subsequently heated from 0 K to 300 K over 100 ps under the NVT ensemble, during which harmonic positional restraints of 2.0 kcal mol^−1^ Å^−2^ were applied to the receptor and ligand. Temperature was maintained using a Langevin thermostat with a collision frequency of 2.0 ps^−1^. This was followed by 14 stages of stepwise equilibration, allowing progressive membrane reorganization around the transmembrane helices while gradually releasing backbone restraints until complete relaxation.

Because GPCR activation is inherently governed by conformational ensembles and rare transitions across metastable states, conventional molecular dynamics alone often remains insufficient to adequately sample activation-related conformational space. We therefore employed Gaussian accelerated molecular dynamics (GaMD) to facilitate exploration of otherwise sparsely populated conformational states by reducing energetic barriers. Dual-boost GaMD (igamd = 3) was implemented to simultaneously accelerate both total potential and dihedral potential energies [42]. Following equilibration, each molecular simulation system underwent 2 ns of conventional MD to collect initial potential energy statistics, followed by 2 ns of GaMD equilibration for adaptive boost-potential calibration. During simulation, the mean and standard deviation of potential energies were updated every 100 ps (50,000 steps), and the upper limit of the standard deviation for both total and dihedral boost potentials was set to 6.0 kcal mol^−1^ (sigma0P/D = 6.0). Production GaMD simulations totaled 6 μs, implemented as six consecutive 1 μs trajectories with continuous state propagation to preserve long-timescale conformational continuity. All simulations were performed at 300 K under the NVT ensemble with a 2 fs timestep, using the SHAKE algorithm to constrain hydrogen-containing covalent bonds. Long-range electrostatic interactions were treated using the particle mesh Ewald (PME) [43,44] method with a nonbonded cutoff distance of 8 Å. Trajectory snapshots were saved every 10 ps for downstream analyses of receptor dynamics, conformational kinetics, and allosteric communication.

### 4.2. Structural and Dynamic Characterization of APLNR

To characterize ligand-induced changes in receptor flexibility and collective motion, structural and dynamic analyses were performed on the production GaMD trajectories of APLNR in the apo and CMF-019-bound states. These analyses were designed to quantify global conformational stability, residue-level flexibility, local packing interactions, and large-scale correlated motions associated with activation-related receptor rearrangements.

Backbone root-mean-square deviation (RMSD) was first calculated relative to the initial structure to evaluate global conformational stability throughout the simulations. Root-mean-square fluctuation (RMSF) analysis was then performed to quantify residue-level mobility and identify regions exhibiting ligand-dependent changes in flexibility. The temporal evolution of total system potential energy was also examined to evaluate the equilibrium behavior of the simulation systems. All trajectory-based structural analyses were performed using CPPTRAJ 6.24.0 in AMBER Tools 25.

To resolve large-scale correlated motions, essential dynamics analysis was performed using principal component analysis (PCA) on receptor Cα atoms [45]. Prior to PCA, trajectories were aligned to the reference structure by least-squares fitting to remove global translational and rotational motions. The covariance matrix of Cα positional fluctuations was constructed using the matrix covar command in CPPTRAJ 6.24.0 and diagonalized using the analyze matrix command. The trajectories were then projected onto the leading principal components to identify dominant collective motion modes. In particular, the first principal component (PC1) [46] was used to characterize the largest-amplitude motion direction and the major conformational displacement patterns of APLNR.

To visualize the spatial distribution and directionality of collective motions, porcupine plots were generated from PCA eigenvectors using PyMOL1.8 together with customized PCA visualization scripts [47]. These plots were used to illustrate residue displacement vectors across transmembrane helices, extracellular loops, and intracellular regions, thereby revealing how agonist binding reorganizes receptor flexibility and collective motion patterns.

To characterize the structural consequences of CMF-019 binding at both global and local levels, several complementary structural descriptors were calculated for the apo and CMF-019-bound states using CPPTRAJ 6.24.0. The radius of gyration (Rg) was calculated to assess the overall compactness and conformational dimensions of the receptor, thereby determining whether ligand binding induced global expansion or compaction. The solvent-accessible surface area (SASA) was calculated to evaluate changes in the overall solvent exposure and global structural organization of APLNR. To characterize ligand-induced local secondary-structure rearrangements, residue-level α-helical propensity was analyzed using the DSSP method implemented in CPPTRAJ 6.24.0. For each residue, α-helical propensity was calculated as the fraction of analyzed trajectory frames in which the residue adopted an α-helical conformation, and the ligand-induced change was quantified as ΔPα = Pα^holo − Pα^apo, where Pα^holo and Pα^apo represent the α-helical propensities of the CMF-019-bound and apo states, respectively. Finally, the R3.50–L6.34 distance was monitored as a structural descriptor of intracellular conformational dynamics, as rearrangements within the intracellular helical core are closely associated with GPCR conformational transitions [48]. The distance was calculated for each trajectory frame using CPPTRAJ 6.24.0 to characterize the dynamic sampling of alternative intracellular configurations during the 6 μs GaMD simulations. Together, Rg and SASA were used to assess global changes in receptor architecture, whereas residue-level α-helical propensity and the R3.50–L6.34 distance were used to characterize local structural remodeling and intracellular conformational dynamics, respectively.

### 4.3. Conformational and Kinetic Landscape Modeling

To characterize the conformational organization and transition kinetics of APLNR in both apo and CMF-019-bound states, Markov state models (MSMs) were constructed from the production trajectories generated by Gaussian accelerated molecular dynamics (GaMD). MSMs provide a framework for discretizing high-dimensional conformational ensembles into metastable states and quantifying the kinetic transitions governing long-timescale receptor dynamics. Given that GPCR activation frequently proceeds through ensembles of intermediate conformations rather than a single structural endpoint, this approach was employed to resolve activation-related conformational states and their kinetic accessibility.

To capture functionally relevant receptor rearrangements, structural features describing activation-associated conformational changes were extracted from the simulation trajectories. Specifically, Cα–Cα distances between residues located in key structural regions, including the intracellular TM3–TM6 distance [49], were selected to represent receptor conformational transitions associated with GPCR activation. The extracted feature space was subsequently subjected to time-lagged independent component analysis (TICA) to identify slow collective motions underlying receptor conformational dynamics. A lag time of 0.1 ns was used for dimensionality reduction [50], and trajectory data were projected onto the first two independent components (IC1 and IC2) for downstream clustering and visualization.

To discretize the conformational ensemble into kinetically meaningful states, the TICA-transformed trajectories were clustered using the K-means algorithm, generating a total of 600 microstates. Model construction was subsequently guided by implied timescale (ITS) convergence analysis, from which a lag time of 2 ns was selected for MSM generation. The resulting microstates were coarse-grained into four macrostates using Perron cluster-cluster analysis (PCCA+) [51], thereby enabling characterization of large-scale conformational transitions and metastable state populations associated with receptor activation. The four macrostates (S1–S4) were defined solely by the PCCA+ partitioning of the kinetic model rather than by manually imposed structural distance thresholds. Their structural characteristics were subsequently interpreted based on representative conformations and activation-related descriptors, particularly the TM3–TM6 distance reflecting the intracellular opening of the receptor.

To ensure model robustness and Markovian behavior, the constructed MSMs were validated using both ITS convergence and Chapman–Kolmogorov (CK) tests [23]. State transition networks were subsequently reconstructed based on macrostate transition probabilities to characterize the dominant conformational pathways sampled by APLNR. In addition, mean first passage time (MFPT) analysis was performed to quantify transition kinetics among metastable states and evaluate how agonist binding reshapes conformational accessibility within the receptor landscape.

All MSM-related analyses, including feature extraction, dimensionality reduction, clustering, model construction, kinetic validation, and MFPT calculations, were performed using the PYEMMA (version 2.5.7) software package [52].

### 4.4. Allosteric Communication Network Inference and Signal Propagation Analysis

To characterize how CMF-019 binding reshapes long-range communication and allosteric signal propagation within APLNR, we employed a Neural Relational Inference (NRI) framework to infer dynamic residue–residue interaction networks from molecular simulation trajectories [25,53]. NRI is a graph-based deep learning approach capable of reconstructing latent interaction networks directly from time-series conformational data, thereby enabling identification of both short-range local couplings and long-range dynamic communication associated with receptor activation. Given that GPCR signaling emerges from collective motions distributed across distant structural regions, this framework was used to capture the dynamic communication architecture underlying agonist-induced receptor reorganization.

Independent 6 μs GaMD trajectories corresponding to the apo and CMF-019-bound states were used for NRI training and inference. Each trajectory originally contained approximately 600,000 frames, which were uniformly downsampled to 5000 frames to reduce computational cost while preserving conformational diversity across the simulation timescale. Receptor conformations were featurized using the three-dimensional coordinates and velocities of receptor Cα atoms, with all positional and velocity features normalized to a maximum absolute value of 1 to standardize model input. To capture long-range conformational correlations over extended timescales, the featurized trajectories were processed into time-lagged sequences of 50 frames, utilizing a dilation interval of 96 frames between consecutive steps within each sequence. A sliding window mechanism with a stride of 1 frame was applied to the starting points, generating highly overlapping samples.

Within the graph neural network architecture, each receptor residue was represented as an individual graph node, while normalized coordinate and velocity features served as node attributes. The NRI model consisted of a variational encoder and a graph-based decoder, in which latent residue–residue interactions were represented as probabilistic graph edges. The encoder inferred the likelihood of dynamic couplings between residue pairs, whereas the decoder propagated messages along inferred edges to predict future conformational states of the receptor. Model optimization was performed by minimizing the evidence lower bound (ELBO), combining a mean squared error (MSE) term for trajectory reconstruction and a Kullback–Leibler (KL) divergence term to regularize the latent graph distribution. Training was conducted using the Adam optimizer [54] with an initial learning rate of 5 × 10^−4^, a batch size of 1, and a learning-rate decay factor of 0.5 applied every 200 epochs. The model exhibiting the lowest validation loss was selected for downstream network inference.

Following model convergence, inferred interaction probabilities were averaged across all trajectory segments to construct interpretable residue–residue interaction matrices, representing the dynamic coupling strength between residue pairs throughout receptor motion. To characterize large-scale communication organization, residues were further classified into predefined structural modules, enabling reconstruction of domain-level communication networks and comparison of global interaction patterns between apo and CMF-019-bound states. Network visualization was performed using Cytoscape (version 3.10.3) [55].

To further determine whether agonist-induced network rewiring promotes effective signal transmission, shortest-path analysis was performed using Dijkstra’s algorithm [56] to characterize dominant allosteric communication routes across the receptor. Particular attention was placed on signal propagation from the N-terminus toward activation-related intracellular regions, including TM6 and ICL2, which are closely associated with GPCR signaling and downstream effector engagement. By comparing path convergence and communication organization between apo and CMF-019-bound states, this analysis enabled evaluation of how CMF-019 binding reshapes information flow and signaling competence within APLNR.

## Data Availability

The original contributions presented in this study are included in the article/Supplementary Materials. Further inquiries can be directed to the corresponding authors.

## Supplementary Materials

The following supporting information can be downloaded at: https://www.mdpi.com/article/10.3390/ijms27177674/s1.
