# Supplementary material for: Agonist Binding Reshapes the Kinetic Landscape and Allosteric Communication of APLNR Toward Activation-Competent States
Source: Int J Mol Sci. 2026 Aug 27;27(17):7674. doi: 10.3390/ijms27177674 (PMC13565920; doi:10.3390/ijms27177674)
Supplement: Supplementary file 1 [file ijms-27-07674-s001.zip › ijms-4472312-supplementary.pdf]

Agonist binding reshapes the kinetic landscape and allosteric communication of APLNR toward activation-competent states

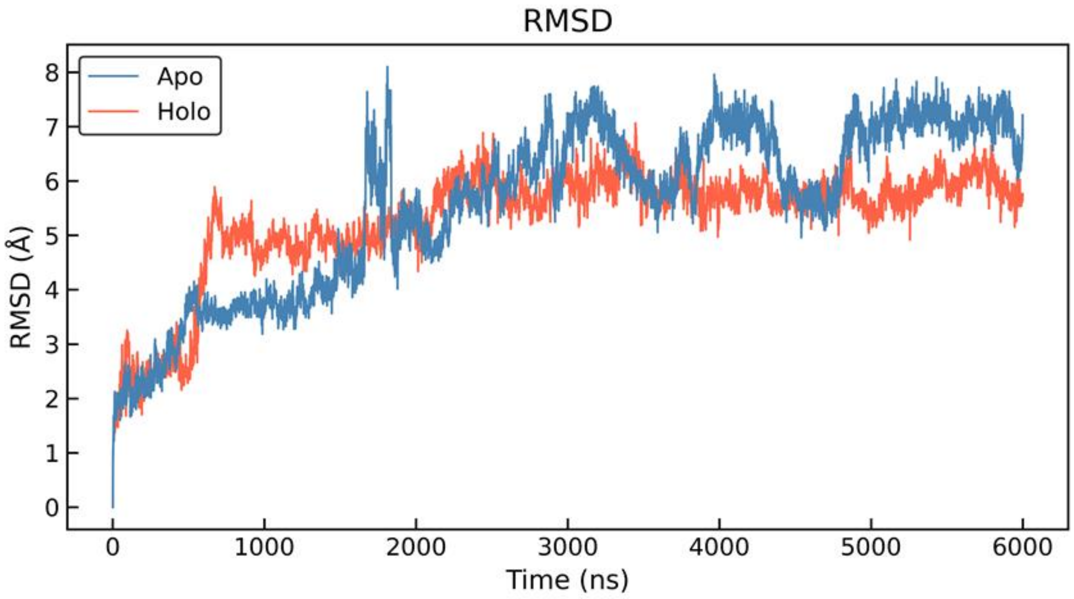

Figure S1. Backbone structural deviation of APLNR during GaMD simulations. Root-mean-square deviation (RMSD) of backbone atoms for the apo (blue) and holo (red) APLNR systems calculated relative to their respective initial structures throughout Gaussian accelerated molecular dynamics (GaMD) simulations.

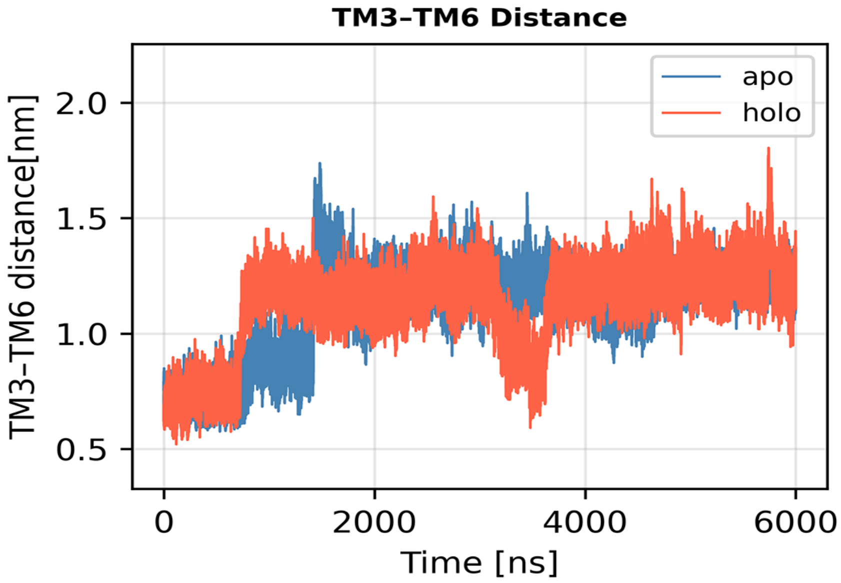

Figure S2. Time evolution of the R3.50–L6.34 distance during the 6 μs GaMD simulations of apo and holo APLNR.

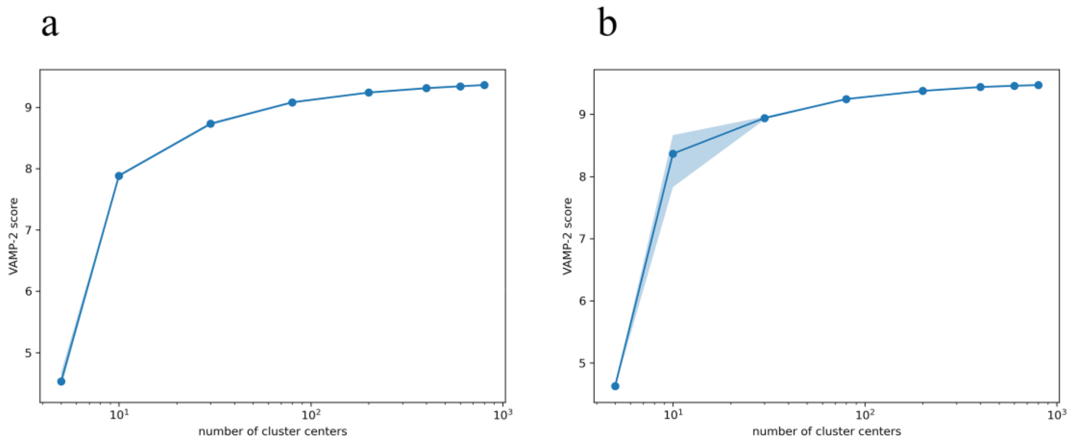

Figure S3. Optimization of microstate discretization based on VAMP-2 scoring. Variational approach for Markov processes (VAMP-2) scores calculated for different numbers of cluster centers used for microstate discretization in the construction of Markov state models (MSMs) for the apo (a) and holo (b) APLNR systems.

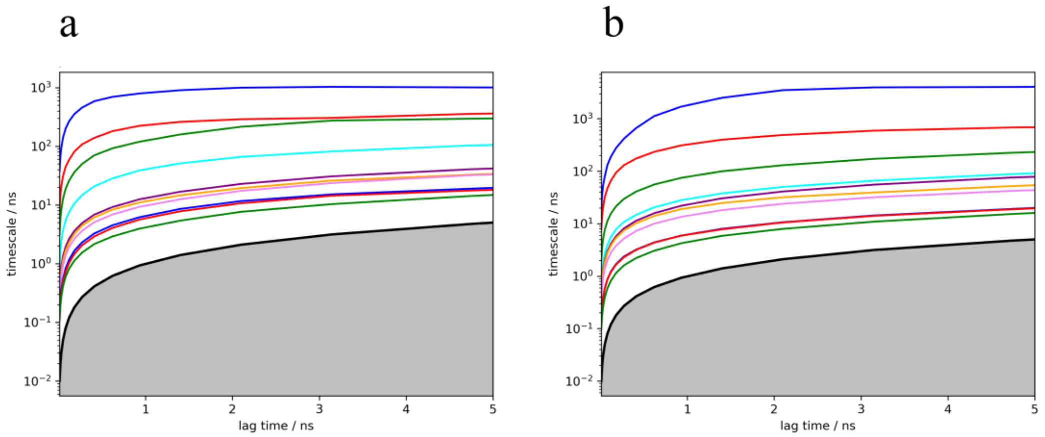

Figure S4. Implied timescale (ITS) analysis for validation of Markov state models. Implied timescales (ITS) calculated as a function of lag time for the construction of Markov state models (MSMs) of APLNR in the apo (a) and holo (b) systems.

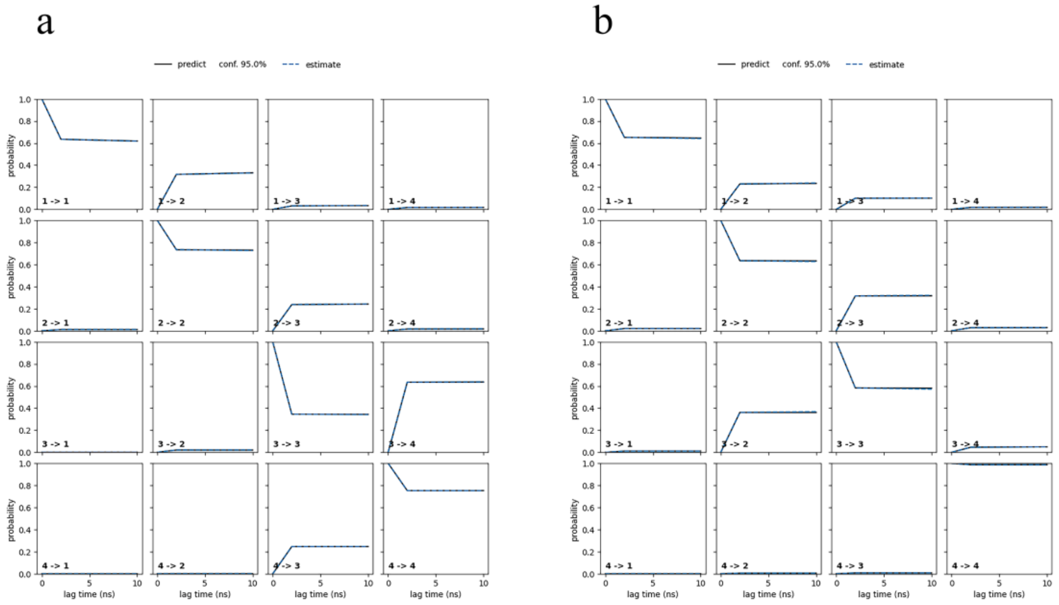

Figure S5. Chapman–Kolmogorov (CK) validation of Markov state models for APLNR. Chapman–Kolmogorov (CK) tests for the Markov state models (MSMs) constructed for the apo (a) and holo (b) APLNR systems. For each metastable state transition, the model-predicted transition probabilities (black solid lines) are compared with transition probabilities directly estimated from simulation data (blue dashed lines) across increasing lag times.
